# Navigating critical time: a qualitative exploration of the experiences of patients and companions in myocardial infarction management

**DOI:** 10.1186/s12872-025-05219-0

**Published:** 2025-10-17

**Authors:** Malihe Imeni, Nahid Dehghan Nayeri, Leila Sayadi, Hossein Ebrahimi, Abbas Mardani

**Affiliations:** 1 Department of Medical Surgical Nursing, School of Nursing and Midwifery, Tehran University of Medical Sciences, Tehran, Iran; 2 Nursing and Midwifery Care Research Center, School of Nursing and Midwifery, Tehran University of Medical Sciences, Tehran, Iran; 3 Center for Health Related Social and Behavioral Sciences Research, Shahroud University of Medical Sciences, Shahroud, Iran; 4 Non-Communicable Diseases Research Center, Research Institute for Prevention of Non-Communicable Diseases, Qazvin University of Medical Sciences, Qazvin, Iran

**Keywords:** Companion, Experience, Myocardial infarction, Patient management, Qualitative study

## Abstract

**Background:**

Myocardial infarction (MI) is an emergency situation, perceived as a unique experience by both the patient and their companion. This study aims to explore the experiences of patients and their companions throughout all stages of myocardial infarction management.

**Method:**

A descriptive qualitative design was employed in this study. Nineteen participants, including 13 patients with a confirmed MI diagnosis and 6 companions, were selected through purposive sampling from a hospital in northeastern Iran. In-person, semi-structured interviews with open-ended questions were conducted, transcribed verbatim, and analyzed using the conventional qualitative content analysis approach.

**Results:**

The study revealed key categories in participants’ experiences before and after their MI. These included “overlooking health risks” due to neglect and misunderstanding of health, and “harmful hesitation” characterized by delays in seeking medical help, often due to denial and self-treatment. Participants also delayed in “understanding the definitive risk”, which led to worsened symptoms. “Missed golden time due to system-level delays” highlighted diagnostic and treatment delays, while a greater “realization of the importance of adherence” to a healthy lifestyle was reported post-MI. The role of companions was also pivotal in influencing patient management and treatment.

**Conclusion:**

Delays in recognizing MI symptoms were influenced by lack of health awareness, hesitation, and healthcare inefficiencies, leading to worsened outcomes. Improving public health education, awareness of MI symptoms, and healthcare responsiveness can reduce these delays. Additionally, the support of companions plays a critical role in ensuring timely medical intervention.

**Supplementary Information:**

The online version contains supplementary material available at 10.1186/s12872-025-05219-0.

## Introduction

Myocardial infarction (MI) is a medical emergency caused by sudden myocardial ischemia due to coronary artery obstruction [1]. Globally, ischemic heart disease (IHD) affects approximately 1,655 individuals per 100,000, with the prevalence projected to exceed 1,845 per 100,000 by 2030 [2]. In contrast, in Iran, the prevalence is slightly lower, estimated at 1,599 per 100,000, and MI accounts for approximately 46% of cardiovascular-related deaths in the country [3, 4].

Timely treatment is crucial in myocardial infarction (MI) care, as it significantly influences recovery rates. Reducing the time between symptom onset and treatment minimizes myocardial damage, improves survival, and lowers complications and mortality [5, 6]. The benefits of reperfusion to damaged tissue depend entirely on timing [7]. The duration from symptom onset to patient treatment is known as total ischemic time (TIT), which predicts disease outcomes [7, 8].

Despite the critical importance of early treatment, research findings highlight significant delays at various stages of TIT. These delays primarily stem from the patient’s decision-making process in seeking medical assistance, transportation to the hospital, time required for disease diagnosis, and initiation of thrombolytic therapy or primary percutaneous coronary intervention (PPCI) [9]. Many MI patients postpone seeking medical assistance for various reasons. A study in Korea found that the time from symptom onset to requesting medical help was 13 h for men and 20 h for women [10]. In Iran, the average delay in hospital visits is 12.9 h, with most patients arriving at least 5 to 6 h late [11, 12]. Such delays often prevent standard therapeutic interventions and can even lead to death [13, 14].

Patients’ awareness of cardiac symptoms and their decision to seek medical help significantly impact treatment delays. Many misinterpret or downplay symptoms, leading to delayed recognition of a MI as a serious condition [15, 16]. On the other hand, studies indicate that interventions aimed at increasing cardiac patients’ knowledge about early treatment have improved awareness but not necessarily behavior [17]. A study in Iran also found that individuals with higher education levels and a history of heart problems tended to seek medical care later [12]. Therefore, exploring patients’ direct experiences and understanding the critical situation from their perspective can help uncover reasons for delays in seeking medical services and provide a basis for addressing these factors.

Delays in MI management are not limited to the pre-hospital period. Despite established protocols, delays persist in diagnosis and treatment phases [12, 18, 19]. According to the American Heart Association, both significant barriers to overcome and opportunities to leverage exist in efforts to improve MI outcomes [20]. Thus, even in centers performing early percutaneous coronary intervention (PCI), reducing TIT remains a challenge [21]. Some studies highlight that, despite visiting treatment centers, healthcare professionals do not always take cardiac symptoms seriously, leading to challenges in receiving timely care [22]. Gaining insight into the experiences of patients and their caregivers throughout the course of an MI episode—starting from symptom onset to hospital care—can help identify barriers to timely intervention and inform improvements in clinical practice.

Identifying the precise causes of delays and improving MI patient management requires a qualitative approach. Achieving the best outcomes in emergency MI care also depends on contextual factors such as cultural beliefs, geographical accessibility, and available resources. In countries like Iran, where diverse cultural norms and urban–rural disparities may influence health-seeking behaviors, such context is especially important. Understanding patient–service provider interactions can enhance early diagnosis and treatment. Accordingly, this study aimed to explore patients’ experiences regarding MI onset, treatment-seeking behavior, and the factors contributing to delays in care within the Iranian healthcare setting.

## Methods

### Design and setting

This research adopted a descriptive qualitative design, using the conventional content analysis approach outlined by Graneheim and Lundman [23]. Qualitative content analysis is a method for the subjective interpretation of textual data, involving a systematic process of coding, as well as identifying themes and patterns. Given the limited number of previous qualitative studies on patients’ and companions’ experiences of MI management, we employed the conventional approach to allow for data-driven insight and avoid imposing preconceived categories. The authors adhered to the standards for reporting qualitative research (SRQR) guidelines for reporting this study (Supplementary file 1) [24].

The study was conducted at a large medical center affiliated with a university of medical sciences, from August 2023 to June 2024. The center, being the largest hospital in northeastern Iran, includes departments for cardiac surgery and angioplasty, serving a substantial number of patients.

### Participants and sampling

The study population included all MI patients visiting a medical center in northeastern Iran. Inclusion criteria consisted of confirmed diagnoses of MI (ST-elevation myocardial infarction [STEMI] and non-ST-elevation myocardial infarction [NSTEMI]) through electrocardiogram (ECG) and laboratory tests by a cardiologist, willingness to participate in the study, ability to communicate and express experiences, age over 18 years, proficiency in Persian, and no history of mental illness. Additionally, patient companions were included when necessary—particularly in cases where patients experienced difficulty recalling or expressing certain aspects of their experience due to the acute nature of their condition. In such cases, companions provided complementary information to support the patient’s narrative. Exclusion criteria included unwillingness to continue participation.

Purposeful sampling with maximum diversity in demographic characteristics (age, gender, education level, marital status, and employment status) was used. After each interview, analysis was conducted, and based on the constant comparative analysis approach and data questioning [25], subsequent participants were added until data saturation was achieved [26].

### Data collection

Data collection began after the study design was approved and ethical clearance was obtained. The researcher visited the cardiac care unit (CCU) of the hospital, reviewed patient files, and consulted with nurses to identify eligible participants. Interviews were conducted in Persian at the hospital. Initially, the study purpose was explained to the participants. Subsequently, patients’ experiences were explored using unstructured and semi-structured in-depth interviews. The interview guide included following questions: (1) Please describe your experiences from the onset of cardiac symptoms to contacting medical staff; (2) What actions did you take to address the symptoms after recognizing them?; (3) What steps were taken for you after contacting emergency medical services (EMS) and arriving at the hospital? To enhance the depth of the interviews, follow-up questions such as “Could you elaborate on that?” and “Can you provide an example?” were used.

With participants’ consent, interviews were recorded. Sampling continued until data saturation was reached, as evidenced by the development of categories and subcategories without the emergence of new findings. A total of 19 participants were included in the study. A researcher-developed questionnaire was used to gather demographic information.

This research was carried out by a team consisting of three faculty members from the nursing and midwifery schools, along with one Ph.D. candidate. The interviewer (MI) brought a wealth of clinical experience working with cardiac patients but had no prior personal relationship with any of the participants. To minimize potential bias, the interviewer maintained reflective notes to consciously recognize and address any preconceptions or assumptions related to the phenomenon being studied.

### Data analysis

The researcher transcribed the recorded interviews verbatim in Persian, and qualitative data management was performed using MAXQDA software, version 12. The analysis was also conducted in Persian to preserve the original meanings and cultural nuances. The research team employed the conventional approach of Graneheim and Lundman [23] for data analysis, following these steps: reviewing the transcribed interviews to ensure a thorough understanding of the content, extracting semantic units and organizing them into condensed units, summarizing and categorizing the condensed units, followed by the selection of appropriate labels, organizing subcategories by comparing similarities and differences among them, and selecting an appropriate title that encapsulated the resulting categories.

For reporting purposes, the categories and selected illustrative quotes were translated into English after the analysis was completed. To minimize any potential loss or distortion of meaning during translation, a bilingual researcher independently reviewed the translated content to ensure conceptual equivalence and clarity.

### Trustworthiness of data

Lincoln and Guba’s criteria—credibility, transferability, dependability, conformability, and authenticity—were employed to enhance the trustworthiness of the data. To establish the dependability of the findings, data were verified by both the participants and the research team. To ensure credibility, the researcher dedicated sufficient time for data collection and returned codes to participants to confirm that the extracted concepts reflected the participants’ original intentions. To enhance transferability, the characteristics and contextual information of participants were described in detail. For confirmability, all data and themes discussed were reviewed with experts during the research process to minimize the influence of the researcher’s perceptions. Authenticity was achieved through continuous reflection and self-criticism throughout the research [23, 27].

### Ethical considerations

This study received ethical approval from the Ethics Committee of Tehran University of Medical Sciences under decree number IR.TUMS.FNM.REC.1402.045. Furthermore, authorization to conduct research within hospital settings and access patient-related information was granted by the relevant healthcare authorities. Prior to participation, all individuals received detailed explanations regarding the study’s purpose, the voluntary nature of their involvement, the assurance of anonymity, their right to withdraw at any stage, and the confidentiality of all collected data. Before initiating data collection, written informed consent was secured from all participants, along with their explicit permission for audio recording of the interviews.

## Results

The participants in this study included 13 males (68%) and 6 females (32%), with 13 participants being patients (10 males and 3 females) and 6 being companions (3 males and 3 females). The age of the patients ranged from 39 to 83 years, while the companions’ ages ranged from 29 to 66 years. In total, 19 interviews were conducted, lasting from 30 to 60 min, with an average duration of 43 min per interview. Demographic information of the participants is detailed in Table 1.

**Table 1:** The participants’ socio-demographic data

	Patients *N* = 13	Companions *N* = 6
Gender	2	4
Female		
Male	11	2
Age (years)	-	1
20–30		
30–40	1	3
40–50	4	1
50–60	4	-
60–70	2	1
70–80	1	-
80–90	1	-
Marital status	-	1
Single		
Married	13	5
Education level	9	-
Under diploma		
Diploma	2	2
Academic	2	4
Occupation	-	3
Health care professional		
Housekeeper	2	-
Retired	4	1
Employee	1	-
Freelance	6	2
History of heart disease	9	
Yes		
No	4	
History of smoking, tobacco, and alcohol use	9	
Yes		
No	4	
Visit to a medical center within the past week due to experiencing cardiac symptoms	6	
Yes		
No	7	
Type of MI	6	
Extensive		
Anterior	5	
Posterior	1	
NSTEMI	1	
Initial treatment	1	
PPCI		
RTP	8	
Other medications	4	

*NSTEMI* Non-ST-Elevation Myocardial Infarction, *PPCI* Primary Percutaneous Coronary Intervention, *RTP* Reperfusion Therapy

**Fig. 1:**
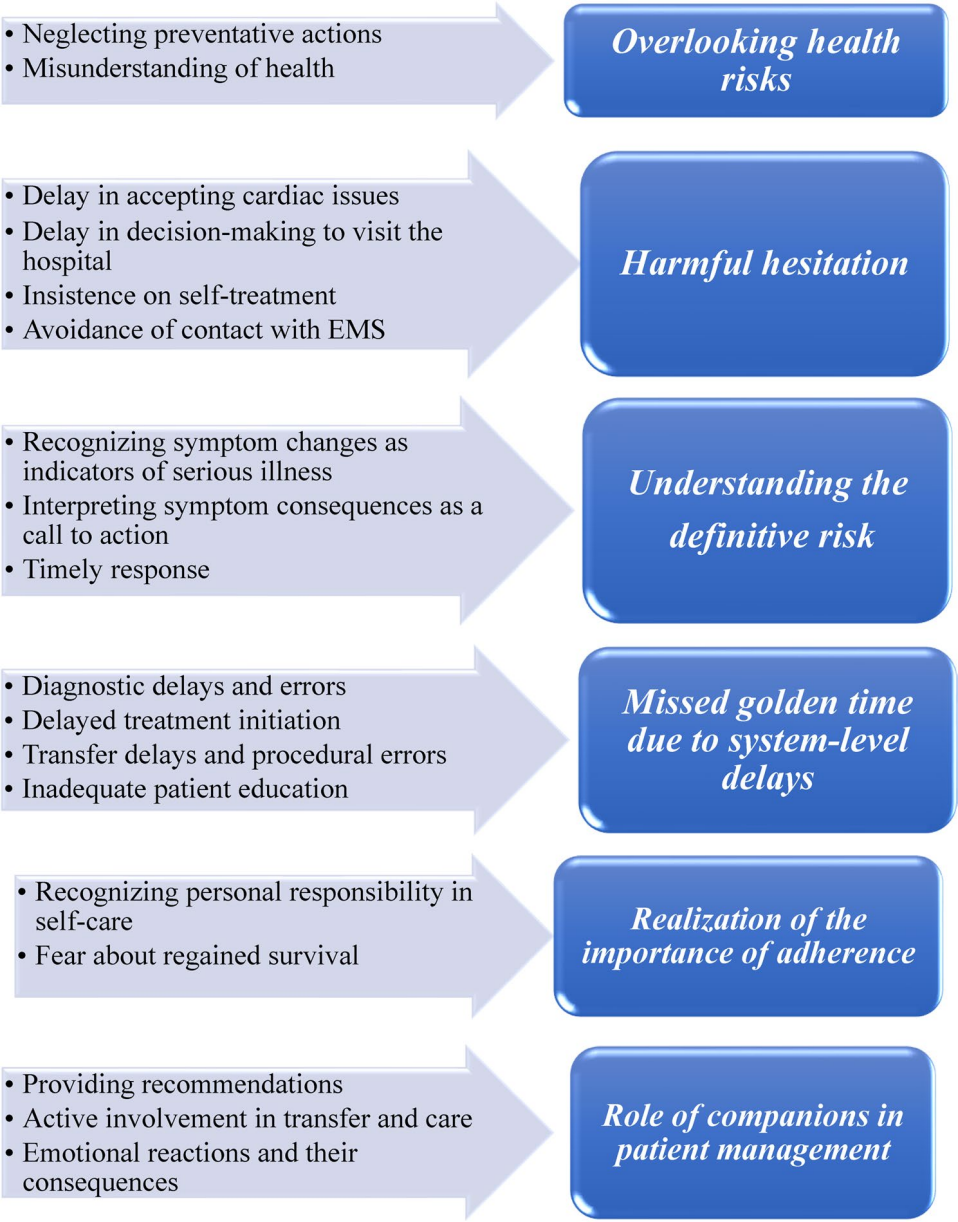
Subcategories and categories developed in this study

Participants reported varied experiences from the moment they noticed changes in their physical condition. In some cases, these changes occurred one or more weeks before the MI. After a thorough analysis of the data, a total of 645 semantic units were identified, which were subsequently merged into 573 unique codes. Ultimately, the resulting codes were categorized into 6 main categories and 18 subcategories. The study findings are summarized in Fig. 1.

### Overlooking health risks

Participants in this study did not engage in activities to assess or improve their health prior to experiencing MI. This pattern of behavior reflects a general lack of attention to preventive measures and misperceptions about their health status. This main category consists of two subcategories: “neglecting preventative actions” and “misunderstanding of health”.

#### Neglecting preventative actions

Most participants did not engage in preventive health behaviors such as regular health monitoring or timely medical consultations. This negligence was manifested through ignoring signs of aging, failing to follow up on physician recommendations, and not monitoring their health status. The underlying reasons varied and included a lack of awareness regarding the importance of prevention, limited health literacy, mistrust in the healthcare system, or a perceived absence of current symptoms.

*“I had never gone to the doctor; this is my first time being hospitalized. Whenever my wife said let’s go for a check-up*,* I didn’t go. Because I was feeling fine and didn’t have any problem until this time”. (Participant (P) 3)*

Some individuals expressed explicit resistance toward medical advice, doubting its necessity or effectiveness.

*“I don’t believe in doctors and taking medication at all. In the past five or six years, I’ve been prescribed blood tests maybe 4 or 5 times, but I haven’t gone even once. I won’t go at all”. (P5)*

Despite receiving specialist recommendations and prescriptions for preventive measures, several patients chose not to follow through, continuing unhealthy lifestyle habits like smoking and poor nutrition.

*“I smoke too*,* and I don’t really watch my diet. Unfortunately, we don’t really take care of ourselves, even though the situation is like this and I should be more careful”. (P5)*

This neglect and resistance sometimes resulted in irreversible consequences. For instance, failure to undergo timely interventions left some patients without viable treatment options.

*“Doctor had prescribed an angiography for me 17 years ago, but I didn’t go. When I finally went for the angiography, they said nothing can be done about it. I mean, the angiography didn’t work”. (P11)*

#### Misunderstanding of health

Participants recognized the importance of following health guidelines but lacked sufficient knowledge on how to apply them, often having misconceptions. This was particularly evident in their understanding of diet and physical activity. For instance, while they knew to limit salt and oil intake, they didn’t realize the importance of restricting other foods like nuts.

*“We really pay attention to our diet; we eat very little oil and try to eat healthy foods as much as possible. For example*,* we consume a lot of nuts.” (P2).*

Additionally, some patients misunderstood appropriate activity levels, either overexerting themselves or not engaging in enough physical activity.

*“I used to walk uphill quickly and felt my arm getting a bit heavy. When I returned home*,* I would do about 45 minutes of stretching and push-ups”. (P2)*

### Harmful hesitation

A substantial delay occurred before patients visited the hospital, often due to ignorance, neglect, or a refusal to acknowledge the severity of their condition. Many patients felt confused and lost control when experiencing symptoms. Initially, they avoided healthcare centers and attempted various self-management methods. Even when they chose to seek medical help, delays in visiting the hospital persisted. This category includes four subcategories: “delay in accepting cardiac issues”, “delay in decision-making to visit a healthcare center”, “insistence on self-treatment”, and “avoidance of contact with emergency medical services (EMS)”.

#### Delay in accepting cardiac issues

Some patients, even after visiting a healthcare center, still did not accept that their issue was related to their cardiovascular system, attributing it to other organs. This delay in acceptance was influenced by a lack of knowledge about cardiac symptoms, absence of specific symptoms, and personal beliefs about heart disease. The lack of awareness about cardiac symptoms led some patients to misinterpret them.

*“I’ve been feeling this heaviness in the middle of my chest for about 6 months to a year*,* but I thought it had to do with my stomach and was just heartburn”. (P4)*

For others, underlying conditions like diabetes masked symptoms, making it harder to recognize heart problems.

*“I didn’t have severe pain at all; it was mostly palpitations that bothered me. When I went to the doctor*,* they said one of my heart arteries was 90% blocked and the other was 60%”. (P6)*

Beliefs about health, such as being physically active or, not having a history of heart disease also contributed to their non-acceptance of the problem.

*“The day I felt heaviness in my chest and had a choking sensation*,* I told my wife that whatever this is*,* it’s not my heart; I’ve never had heart problems before”. (P3)*

#### Delay in decision-making to visit the hospital

Despite patients’ acceptance of their symptoms and acknowledgment of the need to seek hospital care, delays were often influenced by a range of personal, social, and emotional factors rooted in diverse contextual circumstances. These included concerns about family responsibilities, emotional preparedness, healthcare system mistrust, and cultural priorities.

Some participants delayed their decision due to the need for a trusted companion, especially among older adults or those with limited literacy who relied on family members for navigating hospital admission processes.

*“I first called my daughter. She came right away*,* and then she called the ambulance because we couldn’t go to the hospital alone. My wife is disabled and cannot handle administrative tasks and hospital admissions alone*,* so we had to wait for my daughter to arrive”. (P8)*

Others reported emotional and cognitive barriers, such as fear of uncovering a serious diagnosis or a belief that seeking care might worsen their psychological state.

*“I think that now that I’m feeling fine*,* if I go to the doctor*,* they might say something and unsettle me. Right now*,* since I feel fine and don’t know anything, at least I’m at ease”. (P1)*

Cultural context also played a significant role. In Iranian society, where family obligations are a core value, participants delayed hospital visits to avoid alarming family members or disrupting social norms, particularly during family gatherings or guest visits.

*“It was 5:30 AM when I felt pain in my arm again. We had guests over*,* so I thought I’d just step outside for a walk so the others wouldn’t wake up. I felt a sharp pain in my left shoulder*,* and eventually*,* I had to wake my kids and tell them to call an ambulance”. (P5)*

#### Insistence on self-treatment

Before seeking medical care, participants attempted to manage their conditions through various self-treatment methods. Many tried enduring the symptoms or adjusting their daily activities, while others used herbal remedies, prescribed medications, or applied physical pressure to alleviate discomfort.

*“I generally try to visit the doctor not very often; I try to solve it at home. But this time*,* no matter how much I tolerated it*,* I saw it wasn’t helping. I had been forced to go”. (P1)*

Some participants applied pressure or massaged the affected area to relieve pain. Others tolerated their symptoms for as long as possible, believing that they would improve over time, based on previous experiences with similar symptoms.

*“My pain was constant but mild. I kept thinking it was just a stomach ache. I would apply pressure, and it would ease the pain, but yesterday, nothing seemed to help”. (P9)*

Additionally, participants used herbal remedies and prescribed medications, either on their own initiative or as advised by others:

*“Before I intended to come home*,* my chest started to hurt. My wife made me some herbal tea*,* and my pain eased a bit”. (P3)*

#### Avoidance of contact with EMS

More than half of the participants reported choosing to go to the hospital using private transportation rather than contacting EMS. This decision was influenced by several factors, including underestimation of the severity of their condition, misconceptions about the efficiency of EMS, and previous negative experiences or assumptions about delays in ambulance response.

Some participants reported that they did not perceive their symptoms as severe or urgent enough to warrant calling EMS and thus preferred to drive themselves.

*“I drove myself to the clinic because it was close and I didn’t feel that bad”. (P7)*

Others, despite being aware of the potential seriousness of their condition, avoided contacting EMS because they believed private transport would be faster. However, such decisions sometimes resulted in delays due to initial visits to ill-equipped facilities and subsequent transfers.

*“My friend brought me to the hospital. They told me that they didn’t have a cardiologist and that I needed to go to Hospital Y, so they transferred us”. (P10)*

### Understanding the definitive risk

At the onset of clinical symptoms, patients did not feel a sense of danger. It seems that patients needed more evidence to reach certainty regarding the existence of a significant problem. This category includes: “recognizing symptom changes as indicators of serious illness”, “interpreting symptom consequences as a call to action”, and “timely response”.

#### Recognizing symptom changes as indicators of serious illness

Participants evaluated the seriousness of their condition based on noticeable changes in their symptoms, including their persistence, intensification, or the emergence of new or unusual manifestations. The persistence of symptoms—especially when they lasted longer than previous episodes—was a major factor in prompting individuals to seek medical care.

*“I still had chest pain; last time it resolved on its own*,* but this time the pain was less intense, yet I noticed it persisted and was bothering me, so I thought it wouldn’t hurt to go to the clinic”. (P7)*

Exacerbation of symptoms, particularly pain with increasing intensity or different characteristics, led patients to interpret the condition as more serious. They often compared it to their previous experiences and emphasized the unusual nature of the episode.

*“I felt a severe pain radiating to my back*,* chest, and left arm; it was an unusual pain”. (P7)*

The appearance of new symptoms—especially those spreading to multiple areas of the body—was also interpreted as an urgent sign. Some patients delayed help-seeking until such new signs emerged, which altered their perception of the situation.

*“Initially*,* I only felt a choking sensation*,* followed by chest pain. As the pain intensified and spread to both arms and my neck*,* I realized it was serious and asked my wife to call an ambulance”. (P3)*

#### Interpreting symptom consequences as a call to action

Participants did not only rely on the nature of the symptoms themselves but also paid attention to the consequences that followed. When symptoms began to interfere with daily activities or physical functioning, they were perceived as warning signs that required immediate attention.

*“I felt weak and sweaty*,* unable to make it back home. I eventually managed to get home and decided I needed to go to city quickly to figure things out”. (P1)*

Disruption of normal sleep due to symptom severity was another alarming outcome that intensified their perception of the seriousness of their condition.

*“I suddenly woke up from pain. I woke up due to the intensity of the pain and immediately told my wife that she needed to call an ambulance”. (P3)*

#### Timely response

An examination of patients’ actions in response to a MI revealed that some patients confronted their symptoms with greater awareness and sought hospital care before a complete MI occurred. However, due to a lack of diagnosis by the physician, they were discharged from the hospital and later returned with extensive myocardial infarction.

*“I went to triage*,* then saw a clinic doctor who did an ECG and found no issue. They gave me a sedative and ketorolac*,* but my pain didn’t subside”. (P11)*

### Missed golden time due to system-level delays

Based on patient statements, it seems that part of the delay in managing patients is related to the diagnostic and treatment processes. This category includes: “diagnostic delays and errors”, “delayed treatment initiation”, “transfer delays and procedural errors”, and “inadequate patient education”.

#### Diagnostic delays and errors

Despite timely visits to the hospital or contacting EMS, many patients faced delays or errors in the diagnosis of their myocardial infarction (MI). Some patients were discharged without a diagnosis, while others were not transferred to the hospital after contacting EMS. A primary cause of delay was healthcare staff’s focus on diagnostic tests and disregard for the patient’s clinical condition.

*“The doctor reviewed my ECG*,* found no issue*,* gave me sedatives*,* and discharged me. However*,* after walking outside*,* the pain returned*,* so I returned to the hospital and was admitted”. (P11)*

In other cases, symptoms were trivialized, leading to a delay in diagnosis. Some patients were misdiagnosed due to non-specific symptoms or were dismissed based on their history and clinical presentation.

*“I had severe chest pain for three days*,* and a general practitioner prescribed aluminum syrup*,* dismissing it as a stomach issue”. (P9)*

Errors in referral also contributed to delays, with patients either misdirected during triage or not transferred to the hospital despite showing clear signs of a MI.

*“I couldn’t sleep due to pain*,* so my wife called EMS. I reported back and arm pain*,* but they dismissed it as fatigue*,* though I felt something was wrong”. (P10)*

#### Delayed treatment initiation

Some patients experienced delays in the treatment of MI, including PPCI, because the catheterization lab at the study center was only operational during specific hours and was not available 24/7.

*“At the hospital*,* I received injections that eased the pain. After 5–6 hours of rest*,* the pain disappeared by the time I was taken for angiography”. (P5)*

#### Transfer delays and procedural errors

The delay was partly due to the transfer of patients between various departments and medical centers. Additionally, timestamps in the medical records indicate that delays also occurred during interdepartmental transfers within the hospital. Furthermore, there were reported errors in the way patients were transferred.

*“When the ambulance arrived*,* they gave me a sublingual pill along with a few other pills*,* then they took my hand*,* and I walked into the ambulance myself”. (P3)*

#### Inadequate patient education

Patients with symptoms engaged in heavy physical and occupational activities without regard for their physical condition. Despite visiting a physician, they did not receive education on adjusting their activity levels, the necessity of following a diet, and lifestyle modifications. Consequently, they did not change their behaviors and even increased their activity levels.

*“I went to see a specialist because I felt heaviness in my chest. He examined me and said there was no problem. This misled me; I felt relieved that there was nothing wrong*,* and after that*,* I actually increased my exercise intensity because the doctor said there was no issue”. (P2)*

### Realization of the importance of adherence

After experiencing a recent MI, patients acknowledged the importance of adhering to a healthy lifestyle, diet, and medication regimen. This category includes: “recognizing personal responsibility in self-care” and “fear about regained survival”.

#### Recognizing personal responsibility in self-care

After the MI, patients recognized their role in managing their health and mentioned that each individual plays the most crucial role in their own care.

*“I believe everyone should be their own doctor. We must take precautions before something happens; you need to take care of yourself before an event occurs”. (P8)*

#### Fear about regained survival

Participants spoke with fear and doubt about their chances of survival and sustained health after experiencing a MI, mentioning decisions to modify their lifestyle and regularly visit a physician.

*“Honestly*,* I only smoke cigarettes*,* and now I really shouldn’t. The pressure I’ve been under means I have to be much more careful”. (P3)*

### Role of companions in patient management

Companions played a significant role in managing the patient. Their actions and emotions influenced the management process. This category includes three subcategories: “providing recommendations”, “active involvement in transfer and care”, and “emotional reactions and their consequences”.

#### Providing recommendations

Companions almost always provided recommendations to the patient. They also offered advice for controlling and managing symptoms.

*“I got into the car*,* and my heart started hurting again. My wife said you need to rest; you’ll definitely be okay*,* and once you feel better*,* then you can sit behind the wheel”. (P3)*

In some cases, the patient’s companion delayed the management process due to their own circumstances and advised against seeking treatment at a medical center.

*“When I felt the pain*,* I knew it was related to my heart. Despite my wife’s concerns*,* I insisted on going to the hospital because I knew the pain wouldn’t go away at home and wanted to return to her quickly”. (P8)*

#### Active involvement in transfer and care

Since more than 50% of patients arrived at the hospital by private vehicle, companions played a crucial role in transporting patients:

*“I brought my mom to the hospital myself; I quickly put her in the elevator and brought her up”. (P15)*

#### Emotional reactions and their consequences

Companions exhibited reactions and emotions in response to the stressful situations created for the patient, which significantly influenced the patient management process. The most prominent response to the occurrence of a MI was anxiety and panic, making it difficult for the individual to manage the situation correctly and impairing their decision-making.

*“I didn’t even think to call an ambulance. I waited until morning to call my brother after my husband’s pain started at night. At that moment, I didn’t consider what the right action was”. (P12)*

Additionally, some companions faced challenges in persuading the patient to seek medical attention. Companions also reported feelings of guilt and remorse due to the complex and severe condition of the patient.

*“I saw my own shortcomings. I kept thinking that if only I had taken my dad to get an ECG when he said he was feeling bad and mentioned that his heart hurt”. (P12)*

## Discussion

This study explored the experiences of MI patients and their companions at all stages of disease management, even before the onset of cardiac symptoms and visits to medical centers. Since all aspects of patient care were examined, a comprehensive and unique perspective was obtained.

The “overlooking health risks” emerged as a key category in this study, highlighting how patients often neglected preventive measures due to misunderstandings about health. Many failed to monitor their health status, adhere to preventive medications, or maintain a healthy lifestyle, sometimes suffering the consequences. Unlike previous studies that primarily attributed these behaviors to neglect, this research found that patients also acted against their health due to misconceptions, such as excessive physical exertion and obsessive concerns about medication use. Participants expressed regret over these actions, emphasizing the need for improved health literacy. A study indicated significant challenges in health literacy among patients with acute coronary syndrome, reinforcing the importance of providing accurate health information to improve outcomes [28]. Similarly, Brunner et al. found that patients tended to discontinue preventive cardiovascular medications to reduce their medication burden, aligning with the current study [29]. Another study also revealed a preference for taking fewer medications with less frequency [30]. These insights suggest that tailoring medication regimens to fit patients’ daily routines and addressing misconceptions can improve adherence. Moreover, health policies often focus more on disease management than prevention, highlighting the need for further research on health literacy and preventive strategies for cardiovascular patients.

The “harmful hesitation” category highlighted that a significant portion of delays in seeking medical care stemmed from patients’ own reluctance. Many hesitated to acknowledge their cardiac issues, decide on visiting a medical center, or seek timely treatment. Factors such as lack of symptom awareness, nonspecific symptoms, and personal beliefs contributed to denial and delayed action. Even after recognizing their condition, concerns about family, work commitments, and doubts about treatment effectiveness led them to postpone seeking medical help, often managing symptoms with self-care methods and avoiding EMS. Previous studies support these findings, showing that patients frequently delayed medical attention due to symptom misinterpretation, personal obligations, and reluctance to contact EMS [5, 31]. Notably, even nurses and healthcare professionals exhibited similar delays, emphasizing the need for better symptom recognition and timely triage, especially for elderly and diabetic patients with mild or atypical symptoms. Cultural factors also played a role, as many Iranian patients denied their illness to maintain family and professional responsibilities, avoiding acknowledgment of health issues to preserve their sense of integrity.

The “understanding the definitive risk” category highlighted that patients recognized the severity of their condition only after significant symptom changes, such as worsening symptoms, loss of activity, or disrupted sleep. Some delayed seeking medical care even when symptoms worsened, taking action only when basic functions like walking became impossible. For example, in previous studies, patients often waited until experiencing severe changes before seeking help, with chest pain and radiating pain to the left arm being key warning signs [22]. Additionally, other research has shown that patients commonly perceived symptom deterioration as a sign of imminent danger or death [17].

The fourth category identified in the study, “missed golden time due to system-level delays”, highlighted delays not only before visiting the hospital but also during the transfer, diagnosis, and treatment phases. Even patients who contacted the treatment team promptly faced challenges such as misdiagnosis, inappropriate referrals, and delays in diagnostic and therapeutic actions. One significant finding was that patients who minimized their symptoms out of modesty, concern for not burdening others, or cultural humility were often not taken seriously by the treatment team, leading to further delays. These findings align with previous research, which noted diagnostic errors due to complex patient conditions and unusual clinical symptoms. Factors like the knowledge of the treatment team, EMS personnel experience, and the triage process all influenced the diagnosis and care delivery [32]. Other studies also pointed out challenges in accessing healthcare and contacting the treatment team, exacerbated by patients’ unfamiliarity with cardiac symptoms, which could have severe consequences [22]. Addressing issues such as staff shortages, lack of necessary equipment, and inadequate training for EMS personnel may help reduce delays in MI care.

Another category identified in this study was “realization of the importance of adherence”. Most patients, particularly after a MI, recognized the need to adhere to treatment regimens and make lifestyle changes to preserve their health. Having experienced life-threatening situations, they felt fear and uncertainty about their futures. This recognition of adherence and self-care is the first step toward healthier behaviors and treatment compliance. Similarly, a previous study found that MI patients develop a new understanding of self-care and treatment adherence [33]. This shift can be understood through Ryan and Deci’s self-determination theory, which suggests that individuals are more motivated when they feel in control of their choices. Promoting this sense of self-determination can improve adherence, enhancing patients’ quality of life and reducing healthcare costs [33, 34].

The final category identified was “role of companions in patient management”. Companions play a significant role in patient care, sometimes facilitating the process and other times causing delays. In this study, companions were crucial in persuading patients to seek medical attention, with many patients arriving at the hospital by private vehicle. This finding aligns with previous studies, which highlighted the active involvement of family in reducing delays. In the cultural context of Iran, family support is expected during times of crisis. Additionally, periodic screenings can help identify individuals at risk of MI, leading to better management. Companions also play a key role in encouraging treatment adherence, as family support can motivate patients. However, results from a meta-analysis indicated that consulting with family could lead to delays in seeking medical care [35]. Furthermore, family involvement can strengthen adherence to treatment regimens, as emphasized in earlier research [33].

### Limitations

Since the participants in this study were from only one medical center, this limited the transferability of data to other contexts and cultures. Additionally, participants were purposively selected in consultation with a nurse to ensure they met the inclusion criteria and were able to articulate their experiences. Although this approach supported the richness of the data, it may have introduced selection bias and influenced the diversity of perspectives captured. Moreover, the present study was conducted after the occurrence of MI, and future studies can be conducted with an emphasis on understanding preventive processes and assessing individuals’ understanding of MI prevention and health literacy levels to properly identify the structures for preventing such injuries. Furthermore, although rigorous steps were taken to ensure accurate translation of participants’ narratives from Persian to English, including review by a bilingual expert, some nuances may have been lost or slightly altered during the translation process.

## Conclusion

This research explored the experiences of patients and their companions from the onset of a MI through to their interaction with the healthcare system, including the early phases of management. The goal was to identify barriers to timely and effective care and to inform future interventions aimed at improving outcomes. Most patients did not assess their health or take preventive action regarding cardiovascular risk factors prior to experiencing an MI, which may reflect a broader health system emphasis on disease treatment rather than prevention. Many delayed seeking care, attempted self-treatment, or misinterpreted symptoms, particularly when symptoms were non-specific. Such cases were at increased risk of misdiagnosis, emphasizing the need for careful evaluation and triage based on detailed clinical histories. Emergency and triage personnel should be selected and trained to identify even subtle signs of cardiac events. Despite advancements in clinical guidelines and care systems, delays in diagnosis, transfer, and treatment continue to occur. Continuous evaluation and improved coordination across departments are essential for reducing these delays and improving the timely management of MI patients.

## Supplementary Information

Supplementary Material 1.

## Data Availability

Data are available upon reasonable request. The full interviews generated and analyzed during the current study are not publicly available due to individual privacy issues but parts are available from the corresponding author on reasonable request.
